# Management of Acute Onset Catatonia Postpartum in a Patient With a History of Major Depressive Disorder With Psychotic Features: A Case Report

**DOI:** 10.7759/cureus.84392

**Published:** 2025-05-19

**Authors:** Elena Silverstein, Tajudeen O Basiru, Maninder Aulakh, Melissa Verzura, Rogelio Suarez

**Affiliations:** 1 Foundational Sciences, Nova Southeastern University Dr. Kiran C. Patel College of Osteopathic Medicine, Fort Lauderdale, USA; 2 Developmental Behavioral Pediatrics, Dell Children's Medical Center, Austin, USA; 3 Psychiatry and Behavioral Sciences, Community Health of South Florida, Inc., Miami, USA; 4 Psychiatry, Mount Sinai Medical Center, Miami Beach, USA

**Keywords:** catatonia, ect, lorazepam, postpartum, postpartum psychosis

## Abstract

Catatonia is a rare but potentially life-threatening neuropsychiatric syndrome characterized by a range of motor, behavioral, and affective abnormalities. Catatonia is broadly categorized into three clinical presentations: associated with a psychiatric disorder, secondary to a medical condition, or of unspecified origin. Postpartum catatonia remains poorly understood, particularly in cases where there is no active psychiatric or medical illness at the time of onset. Most documented cases occur in the context of severe peripartum mood or psychotic episodes, highlighting the unique nature of catatonia emerging independently in the postpartum period.

In this case report, we present a 33-year-old woman with a known history of major depressive disorder with psychotic features who developed catatonia within 24 hours following childbirth. Notably, the patient exhibited no signs or symptoms of depression or psychosis during the pregnancy or at the time of catatonic onset. She denied experiencing any mood or psychotic symptoms leading up to delivery, and her presentation was not consistent with a relapse of her previous psychiatric condition. This case is notable for its unique context: catatonia emerging in the immediate postpartum period without concurrent psychiatric decompensation or identifiable medical triggers. It raises important clinical questions about whether postpartum physiological or hormonal changes alone may precipitate catatonia in vulnerable individuals, even in the absence of overt psychiatric illness.

Our report underscores the importance of maintaining a high index of suspicion for catatonia in postpartum patients, including those without active psychiatric symptoms. Early recognition and treatment are critical for optimal outcomes. Furthermore, this case highlights the need for more research to better understand the spectrum of postpartum catatonia and to determine whether it may, in some instances, represent a distinct clinical entity separate from traditional psychiatric or medical frameworks.

## Introduction

Catatonia is a rare behavioral syndrome characterized by psychomotor disturbances such as stupor, mutism, rigidity, posturing, echolalia, and echopraxia [1]. Although uncommon, catatonia can occur in the postpartum period - a complication that remains severely understudied due to its rarity [1]. Following childbirth, women experience rapid hormonal changes: estrogen and progesterone levels drop sharply, while oxytocin and prolactin increase to support uterine contraction and breastfeeding initiation [2]. These dramatic physiological shifts may contribute to the development of catatonia, though this connection is not well understood. Postpartum catatonia is often associated with underlying psychiatric disorders - in fact, 20% of patients with postpartum psychosis will develop catatonia, but its pathophysiology remains unclear [3]. The exact timing of onset in the postpartum period is also unknown due to limited research [1]. However, postpartum catatonia presents with symptoms similar to those seen in the general population and responds to the same treatment approaches [1].

Catatonia is a neuropsychiatric syndrome characterized by various motor, behavioral, and emotional abnormalities [4]. It can occur with psychiatric conditions like mood disorders, schizophrenia, and psychosis, or due to medical issues such as infections and metabolic problems [4]. Catatonia involves problems in brain motor circuits, leading to both reduced and excessive movement [5]. Studies suggest it affects circuits that control movement initiation, organization, and speed [5]. Brain imaging shows reduced activity in areas like the supplementary motor area (SMA), primary motor cortex, parietal cortex, and basal ganglia during voluntary movements [5]. During catatonic episodes, however, the SMA and M1 can become overactive, possibly explaining movement issues [5]. Other findings include abnormal connections between motor regions and delayed movement signals, which may stem from disrupted inhibition involving the basal ganglia and thalamus. Overall, research highlights motor system dysfunction as central to catatonia [5].

The diagnosis of catatonia relies on careful clinical evaluation and recognition of characteristic motor and behavioral symptoms [6]. Diagnostic criteria, such as those outlined in the Diagnostic and Statistical Manual of Mental Disorders, Fifth Edition (DSM-5) or the Bush-Francis Catatonia Rating Scale, provide structured frameworks for assessment. Key features include at least three of the following symptoms: immobility (stupor), mutism, posturing, waxy flexibility, negativism, echolalia, echopraxia, or stereotypy [6]. A thorough history and physical examination, along with diagnostic tests to exclude medical causes, are essential in establishing the diagnosis [4]. The "lorazepam challenge," where a significant improvement in symptoms follows the administration of lorazepam, can also be a useful diagnostic and therapeutic tool [4].

Treatment of catatonia is highly effective when initiated promptly, with benzodiazepines such as lorazepam being the first-line therapy [4]. A rapid response to benzodiazepines is often seen quickly, even within minutes, and about 70-80% of patients achieve full remission with benzodiazepines within six days [4,7,8]. If symptoms persist or are severe, electroconvulsive therapy is an effective second-line treatment, with success rates exceeding 80% [9]. Early recognition and treatment are crucial, as catatonia can lead to severe complications, including malnutrition, infections, and even death if left untreated [10]. Prompt intervention significantly improves outcomes, underscoring the importance of early diagnosis and comprehensive care [10].

The postpartum period is associated with a range of psychiatric and physiologic challenges; however, acute catatonia is rarely reported as a primary diagnosis. It is more commonly observed in the context of underlying psychiatric conditions such as postpartum depression, bipolar disorder, or schizophrenia [3,11]. Catatonia in the postpartum period is typically linked to mood or psychotic disorders rather than occurring independently. In this case report, we present a patient with a history of major depressive disorder, but who did not meet criteria for a current depressive episode at the time of presentation. She exhibited no persistent depressed mood, anhedonia, or other hallmark symptoms of a depressive or psychotic episode. Instead, she presented with acute onset postpartum catatonia, independent of other psychiatric or medical conditions, highlighting a rare and atypical clinical presentation.

## Case presentation

This is a case of a 33-year-old G1P0 female with a past psychiatric history of major depressive disorder with psychotic features who presented to labor and delivery at 40 weeks and three days’ gestation, having contractions, and proceeded to have an uncomplicated vaginal delivery. Within hours of her delivery, the patient started to appear withdrawn and nonverbal-only answering questions with nods or simple "yes" or "no" responses. The next day, psychiatry was consulted. At the time the patient was seen, 24 hours after her delivery, she was not responding to verbal or physical stimuli. Relatives and nursing staff denied any reports of anxiety, perceptual disturbances such as auditory, tactile, or visual hallucinations, delusions, depression, or any other mood disorder symptoms.

The patient has a past psychiatric history of major depressive disorder with psychotic features. She was first diagnosed in 2016 when she was admitted to a psychiatric hospital for three days due to severe depression associated with mood-congruent hallucinations and delusions. At this time, she was placed on 50 mg of sertraline daily, administered orally, and 1 mg of risperidone daily, administered orally, and achieved remission. In 2020, the patient began to taper off her risperidone and had a recurrence of her visual hallucinations, paranoia, and depression. After this recurrence, she restarted her risperidone. Again, in 2022, the patient stopped taking her risperidone and had another recurrence of her symptoms. At this point, she was stable on her regimen and was consistently following up with a psychiatrist and therapist. Once the patient became pregnant, she decided to discontinue her medications for the well-being of the child, although she was counseled on the risks and benefits to both her and her unborn child of discontinuing her medications. During pregnancy, she continued to follow up with her psychiatrist and remained stable. The patient has a strong support system, including her mother and husband, and holds a job in the healthcare field. She does not use alcohol, drugs, or tobacco. She has no history of suicide attempts or self-harm. She has no significant past medical history.

Upon entering the room, the patient made eye contact with the psychiatry team but could not answer any questions verbally or with a nod. Her limbs were limp, and she was unable to hold her hands up on her own, would not blink on command, and did not respond to questions. The patient was in a stupor, mute, and presented with an anxious affect. She was staring at the examiners with minimal blinking. She appeared to be stuck in an indecisive, hesitant motor movement. At this time, the Bush-Francis Catatonia Rating Scale was administered. The patient was given a severity score of 12 and a screening score of 9. The scoring criteria are included in Table 1.

**Table 1:** Summary of findings based on the Bush-Francis Catatonia Rating Scale (BFCRS) Credit: Adapted from [12]

Parameters	Rating
Screening score	9 (1-14)
Severity score	12 (1-23)
Positive items	
Immobility/stupor	2 (0-3)
Mutism	3 (0-3)
Staring	2 (0-3)
Withdrawal	1 (0-3)
Excitement	1 (0-3)
Motorically stuck (ambitendency)	3 (0-3)
Negative items	
Posturing/catalepsy	0 (0-3)
Grimacing	0 (0-3)
Echopraxia/echolalia	0 (0-2)
Stereotypy	0 (0-3)
Mannerisms	0 (0-3)
Verbigeration	0 (0-3)
Rigidity	0 (0-3)
Negativism	0 (0-3)
Waxy flexibility	0 (0-3)
Impulsivity	0 (0-3)
Automatic obedience	0 (0-3)
Mitgehen	0 (0-3)
Gegenhalten	0 (0-3)
Grasp reflex	0 (0-3)
Perseveration	0 (0-3)
Combativeness	0 (0-3)
Autonomic abnormality	0 (0-3)

Given that the patient was in a catatonic state, she was given an intramuscular (IM) dose of 2 mg lorazepam (Ativan) as a challenge. Notably, this dose was given IM, as she did not have IV access, and she was unable to swallow. Improvements were gradual following the administration of 2 mg lorazepam; however, notably, the patient was able to respond with "yes" or "no" answers three hours later. Due to mild to moderate improvements, 1 mg lorazepam (Ativan) three times daily, administered orally, was recommended, and the first dose was given that evening. The patient was not assessed overnight to prioritize rest following childbirth. The next day, when she was seen by the psychiatry team, she was walking, urinating on her own, taking care of her baby, and folding laundry. She was able to recall the previous day's interview and her inability to respond to questions. At this time, the patient continued to deny symptoms of anxiety, perceptual disturbances such as auditory, tactile, or visual hallucinations, delusions, depression, or any other mood disorder symptoms. She was started on her pre-pregnancy dose of sertraline, 50 mg daily, administered orally.

During the interview, the patient was alert and oriented to time, place, and person. She denied suicidal and homicidal ideation, visual and auditory hallucinations, feelings of anxiety, mania, and depression. She reported her biographical information and psychosocial history with accuracy. Psychiatry cleared the patient for discharge. The team attempted to discharge her on 1 mg of lorazepam three times daily, administered orally; however, she refused, stating that it made her feel too sedated. She explained that she did not want to miss bonding time with her newborn. She was, however, educated on how the medication works and the risks associated with incomplete treatment. Following extensive counseling and education on the importance of continuing the treatment plan, she agreed to take 0.5 mg three times daily, administered orally, until follow-up with her psychiatrist. Additionally, the patient was advised not to breastfeed due to the risk of benzodiazepine transmission through breast milk. While selective serotonin reuptake inhibitors (SSRIs) are generally considered safe during breastfeeding, the addition of lorazepam warranted the recommendation to bottle-feed instead [13].

Since discharge, the patient has experienced a gradual recurrence of depressive and psychotic symptoms, which she reported during a follow-up visit with her psychiatrist one month postpartum. She described loss of energy and non-threatening, non-distressing visual disturbances. Importantly, there has been no relapse of her prior catatonia. Risperidone was reinitiated, and the patient remains in good health. The baby is also doing well.

## Discussion

In the DSM-5, catatonia is categorized into three groups: (1) catatonia associated with another mental disorder (e.g., neurodevelopmental disorders, psychosis, bipolar disorder, depression, or other mental illnesses); (2) catatonia due to another medical condition (e.g., encephalitis, traumatic brain injury, or other medical issues); and (3) unspecified catatonia. In this 33-year-old female, the cause of catatonia is currently unknown. Our first differential diagnosis was catatonia associated with major depressive disorder, and our differential diagnosis includes catatonia associated with psychosis or due to a medical condition. The diagnosis of catatonia associated with major depressive disorder is supported by the patient's history of major depressive disorder (MDD) with psychotic features, and she was treated with sertraline and risperidone before her pregnancy. Given her history of MDD and the fact that she was not taking her maintenance medications, sertraline and risperidone, at the onset of catatonia, depression is the most likely diagnosis. However, this diagnosis is complicated by the fact that the patient was not experiencing depressive symptoms when catatonia began.

Psychosis-associated catatonia is less likely, as the patient denies hallucinations, delusions, or other psychotic symptoms such as disorganized speech, disorganized behavior, or negative symptoms before and after the catatonic episode. Lastly, catatonia due to a medical condition is also plausible, as childbirth imposes significant physiological stress, including trauma, hormonal shifts, and hemodynamic changes, which can precipitate medical complications [10]. Overall, the evidence most strongly supports a diagnosis of catatonia associated with depression, particularly given the patient’s psychiatric history and untreated status at onset. It is also notable that pregnancy can act as a protective factor for some mental health conditions, with increased vulnerability during the postpartum period due to hormonal and physiological changes [14]. Ultimately, regardless of the underlying cause of the catatonia, treatment with lorazepam (Ativan) was successful, starting with an initial dose of 2 mg orally, followed by 1 mg three times daily orally. This case highlights an important teaching point: greater awareness and education are needed among healthcare providers regarding the diagnosis and management of catatonia in the postpartum period. A high index of suspicion is crucial to ensure prompt diagnosis and treatment, as delays can significantly impact outcomes. Additionally, social support is essential during this already challenging postpartum phase, especially given the need for strict adherence to frequent and timely medication administration.

An important learning point from this case is that a minimal initial response to a lorazepam challenge should not discourage clinicians from continuing lorazepam treatment for at least one week [15]. A lack of significant improvement during this period may serve as a clinical indication for considering electroconvulsive therapy. Additionally, providing comprehensive patient education is critical to ensure adherence to the treatment plan. In this case, our patient was hesitant to continue lorazepam treatment after the first two to three doses. However, evidence suggests that insufficient treatment may lead to a relapse into catatonia [16].

Importantly, there is a need for further study to examine whether catatonia is a distinct clinical entity, as various authors have called for its designation as a clinical entity of its own [11]. While postpartum psychosis is well documented and characterized by symptoms such as delusions, hallucinations, and confusion, catatonia is generally not considered a standalone symptom or disorder [17]. Instead, it may be a secondary feature of psychosis or mood disorders that manifest during or after childbirth. Some isolated case reports have described catatonia occurring postpartum, but these cases are rare and often involve comorbid conditions such as schizoaffective disorder or bipolar disorder [1]. The occurrence of catatonia immediately after childbirth is not considered typical and is often seen within the broader context of these underlying psychiatric disorders. Furthermore, there is evidence suggesting that continuation of prophylactic medication during pregnancy can reduce the risk of postpartum psychiatric episodes. There is no direct evidence comparing the incidence of postpartum catatonia based on medication status during pregnancy [18]. Additional research is needed to explore this specific aspect.

The case presentation suggests that catatonia may represent a distinct clinical entity, potentially triggered by underlying physiological or psychological stressors. While traditionally associated with psychiatric or medical illnesses, this case raises the possibility that catatonia can, in certain circumstances, manifest without an identifiable underlying condition. Beyond the patient’s history of MDD with psychosis, this patient does not have any known factors predisposing her to catatonia, which is why this case is so interesting. This challenges conventional diagnostic frameworks and highlights the need for clinicians to maintain a high index of suspicion for catatonia, even in the absence of overt psychiatric or medical pathology. Further research is warranted to better understand the underlying mechanisms and to refine diagnostic criteria for cases that fall outside traditional presentations.

## Conclusions

The case of a 33-year-old woman with postpartum catatonia highlights the critical need for timely and proactive psychiatric care during the postpartum period. Ensuring the well-being of both mother and baby requires maintaining a high index of suspicion for catatonia, particularly in patients with a psychiatric history who warrant close follow-up with their psychiatrist. Additionally, educating healthcare providers, including obstetric teams, on recognizing early signs of postpartum catatonia, especially in high-risk patients, can facilitate earlier intervention and significantly improve outcomes. While catatonia can occur in the context of postpartum psychiatric disorders, there is limited evidence to support isolated acute postpartum catatonia as a distinct clinical entity. It is most commonly associated with mood and psychotic disorders, and its postpartum presentation remains rare. Further research is needed to refine diagnostic criteria and better understand the pathophysiology of catatonia in this context.
